# Cortical Electrical Stimulation Ameliorates Traumatic Brain Injury-Induced Sensorimotor and Cognitive Deficits in Rats

**DOI:** 10.3389/fncir.2021.693073

**Published:** 2021-06-14

**Authors:** Chi-Wei Kuo, Ming-Yuan Chang, Hui-Hua Liu, Xiao-Kuo He, Shu-Yen Chan, Ying-Zu Huang, Chih-Wei Peng, Pi-Kai Chang, Chien-Yuan Pan, Tsung-Hsun Hsieh

**Affiliations:** ^1^Department of Life Science, National Taiwan University, Taipei, Taiwan; ^2^School of Physical Therapy and Graduate Institute of Rehabilitation Science, Chang Gung University, Taoyuan, Taiwan; ^3^Division of Neurosurgery, Department of Surgery, Min-Sheng General Hospital, Taoyuan, Taiwan; ^4^Department of Early Childhood and Family Educare, Chung Chou University of Science and Technology, Yuanlin, Taiwan; ^5^Sun Yat-sen Memorial Hospital, Sun Yat-sen University, Guangzhou, China; ^6^Fifth Hospital of Xiamen, Xiamen, China; ^7^Fujian University of Traditional Chinese Medicine, Fuzhou, China; ^8^Department of Internal Medicine, Far Eastern Memorial Hospital, New Taipei, Taiwan; ^9^College of Medicine, Taipei Medical University, Taipei, Taiwan; ^10^Department of Neurology, Chang Gung Memorial Hospital and Chang Gung University College of Medicine, Taoyuan, Taiwan; ^11^Neuroscience Research Center, Chang Gung Memorial Hospital, Taoyuan, Taiwan; ^12^School of Biomedical Engineering, College of Biomedical Engineering, Taipei Medical University, Taipei, Taiwan; ^13^School of Medicine, College of Medicine, Chang Gung University, Taoyuan, Taiwan; ^14^Healthy Aging Research Center, Chang Gung University, Taoyuan, Taiwan

**Keywords:** traumatic brain injury (TBI), cortical electrical stimulation, neuromodulation, sensorimotor impairment, cognitive dysfunction

## Abstract

**Objective**: Individuals with different severities of traumatic brain injury (TBI) often suffer long-lasting motor, sensory, neurological, or cognitive disturbances. To date, no neuromodulation-based therapies have been used to manage the functional deficits associated with TBI. Cortical electrical stimulation (CES) has been increasingly developed for modulating brain plasticity and is considered to have therapeutic potential in TBI. However, the therapeutic value of such a technique for TBI is still unclear. Accordingly, an animal model of this disease would be helpful for mechanistic insight into using CES as a novel treatment approach in TBI. The current study aims to apply a novel CES scheme with a theta-burst stimulation (TBS) protocol to identify the therapeutic potential of CES in a weight drop-induced rat model of TBI.

**Methods**: TBI rats were divided into the sham CES treatment group and CES treatment group. Following early and long-term CES intervention (starting 24 h after TBI, 1 session/day, 5 days/week) in awake TBI animals for a total of 4 weeks, the effects of CES on the modified neurological severity score (mNSS), sensorimotor and cognitive behaviors and neuroinflammatory changes were identified.

**Results**: We found that the 4-week CES intervention significantly alleviated the TBI-induced neurological, sensorimotor, and cognitive deficits in locomotor activity, sensory and recognition memory. Immunohistochemically, we found that CES mitigated the glial fibrillary acidic protein (GFAP) activation in the hippocampus.

**Conclusion**: These findings suggest that CES has significant benefits in alleviating TBI-related symptoms and represents a promising treatment for TBI.

## Introduction

Traumatic brain injury (TBI) is a common brain injury caused by an external mechanical force, such as rapid acceleration or deceleration impact, crushing, or projectile penetration (Faul et al., 2010). TBI has been estimated to affect approximately 1.7 million American residents, resulting in the cost of over $76.5 billion to the medical care systems each year in the United States (Faul et al., 2010). Following TBI, damage to the brain can be identified as primary injury and secondary injury. Primary injury is direct damage to the intracranial contents resulting from mechanical forces, such as an object, rapid acceleration/deceleration, as seen in motor vehicle accidents, penetrating injury, and blast waves. Acute injury of the parenchyma can manifest as contusions, hematomas, shearing of white matter tracts, and cerebral edema (Popernack et al., 2015). Secondary injury is the subsequent damage that occurs over hours to days and results in the alternation of cerebral blood flow and inflammatory processes. In addition, cerebral blood flow is often altered and causes vasospasm, focal microvascular occlusion, and vascular injury, resulting in brain edema. This secondary ischemia can lead to hypoxia and neuronal hyperactivity or excessive inhibition (Ping and Jin, 2016). Individuals with different severities of TBI suffer long-lasting motor, sensory, neurological, cognitive, or behavioral disturbances. To date, no neuromodulation-based therapies have been used to manage the development of pathological deficits associated with TBI.

A number of alternative nonpharmacological procedures have been suggested as new therapeutic strategies for neurological disorders, including TBI. Electrical or magnetic neuromodulation approaches are promising tools for inducing changes in neural activity and plasticity. Repetitive transcranial magnetical stimulation (rTMS) or cortical electrical stimulation (CES) are used as neuromodulatory means for neurological disorders. Recent research suggests that rTMS or CES can alter the neural activities *via* plasticity-like mechanisms, which have been applied for the treatment of neurological or psychiatric disorders and might have the therapeutic potential for TBI (Gaynes et al., 2014; Kamble et al., 2014; Sokal et al., 2019; Zaninotto et al., 2019; Pink et al., 2021). However, the results exploring the therapeutic effects of such neuromodulatory tools on TBI are still inconclusive. The major concern with rTMS is the risk of seizure-induction (Cavinato et al., 2012; Dhaliwal et al., 2015). Under this consideration, individuals with TBI are frequently excluded from rTMS studies making it difficult to assess the efficacy and safety of rTMS as a treatment for TBI (Rossi et al., 2009).

Recently, increasing attention has focused on cortical electrical stimulation (CES). This technique was initially used as an experimental treatment to control neuropathic and intractable central pain (Son et al., 2006; Fagundes-Pereyra et al., 2010; Alm and Dreimanis, 2013). It has been reported that the CES can improve motor and sensory functions in stroke patients (Brown and Pilitsis, 2005). Similar to the rTMS, a recent animal study suggests that CES can modulate motor cortical excitability *via* plasticity-like mechanisms (Hsieh et al., 2015a). Earlier preclinical studies also show that CES coupled with motor rehabilitative training promotes synaptic plasticity and improves motor function after ischemic stroke (Adkins-Muir and Jones, 2003; Adkins et al., 2006, 2008). However, the therapeutic value of such a stimulatory approach for TBI is still unclear.

For the purpose of translational research, an animal model of disease could be the best way to study the pathogenesis of TBI, as it may provide a stable condition to eliminate any discrepancies and clarify the existence of a treatment effect. A suitable TBI animal model could help explore an effective therapeutic strategy, allowing the rapid screening of a stimulation protocol and identifying the detailed mechanisms of the CES protocol in TBI animal studies through neurophysiological and molecular analysis. Although the CES methodology has been reported in a few animal studies, studies with the application of CES as a long-term treatment in TBI animals are relatively rare. To date, the long-term effects of CES on detailed TBI-related motor and nonmotor symptoms, as well as its neuroprotective effects, have not been studied in TBI animal models. The current study was, therefore, designed to identify the therapeutic effects of CES in rats with weight drop-induced TBI. The therapeutic effects of CES were measured by behavioral assessments, including detailed time-course analysis of motor and nonmotor symptoms, such as the modified neurological severity score (mNSS), adhesion removal test, beam walking, novel object recognition (NOR), and histological assessment. It is hypothesized that long-term CES treatment may result in the improvement of TBI-related motor and nonmotor symptoms in weight drop-induced TBI rats. The knowledge obtained in these procedures may have translational relevance for establishing new therapeutic applications as neuromodulation therapy in clinical use.

## Materials and Methods

### Animal Preparation

Experiments were carried out on male Sprague-Dawley rats (350–400 g) obtained from the Animal Center of Chang Gung University. The rats were housed in standard cages in a temperature (25°C) and humidity (50%) controlled facility with a 12 h light/dark cycle. All animal assessment and surgical procedures were approved by the guidelines of the Institutional Animal Care and Use Committee at Chang Gung University (IACUC Approval No. CGU107-104). All efforts were made to minimize the number of rats required in the present study.

### TBI Rat Model

**Figure 1:**
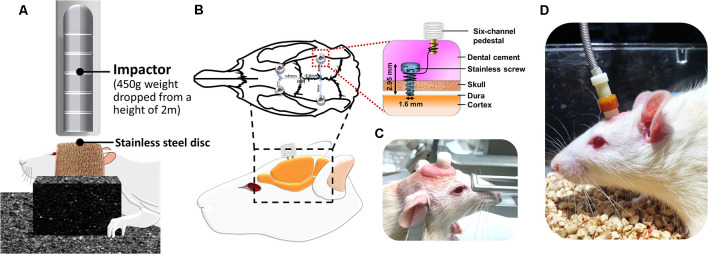
Instrumentation setup of the traumatic brain injury (TBI) rat model and the placement and assembly of the cortical electrical stimulation (CES) electrode. **(A)** TBI rat model was induced using a 450-g brass weight falling from 2 m through a vertical transparent Plexiglas tube. **(B,C)** The CES electrodes were positioned at the frontal and parietal cortex and connected with wires in the six-channel pedestal and fixed with dental cement. **(D)** During CES treatment, the head electrode pedestal served as the plugin site of the electrode pin to conduct electrical current *via* the screw electrode on the cortex.

To provide a stable and controllable environment and obtain detailed mechanical insights from the TBI animal model, researchers have widely used one of the typical TBI rodent models, known as the weight-drop model, to mimic diffuse axonal injury and concussion caused by falls or motor vehicle accidents in individuals with TBI (Foda and Marmarou, 1994; Marmarou et al., 1994). The weight drop model is the use of weights that are freely dropped or through a guiding tube to generate an impact on the head. The widely recognized weight drop induced TBI model is Marmarou’s impact acceleration model, which has been described as resulting in diffused brain injury in rats (Marmarou et al., 1994; Smith et al., 2015). In this model, weight-drop procedures can provide a secure and inexpensive method for producing different graded brain injuries in animals by adjusting the height during the weight drop (1–2 m; Foda and Marmarou, 1994; Marmarou et al., 1994; Hsieh et al., 2017). In the current study, for the induction of TBI in rats, the Marmarou’s impact acceleration model was modified and applied. Animal preparations for the induction of the TBI rat model were described previously (Hsieh et al., 2017). Briefly, to minimize animal suffering and distress during TBI lesions, we anesthetized the animals using an intraperitoneal administration of tiletamine-zolazepam (50 mg/kg, i.p.; Zoletil, Vibac, France) with xylazine (10 mg/kg; Rompun, Bayer, Leverkusen, Germany) 30 min before impact. A 2-cm incision was made, and the area was carefully cleared to expose the line of bregma. The stainless steel disc (10 mm in diameter, 3 mm in thickness) was fixed with the self-adherent wrap (1582, 3M, St. Paul, MN, USA) to the central portion of the rat skull vault between the bregma and lambdoid sutures. The rats were placed prone on flexible foam and secured by using two belts. A Plexiglas tube was then positioned vertically, and the lower end of the tube was centered directly above the stainless steel disc. TBI was induced using a 450-g brass weight falling from 2 m through a vertical transparent Plexiglas tube (Figure 1A). Based on our earlier study, the averaged response of impact force and acceleration are 9.43 ± 0.27 kg and 370.28 ± 9.98 g (Hsieh et al., 2017). Under this weight drop TBI model, the different graded severity of brain injury can be reproducibly and reliably induced. Following the TBI lesion, the body temperature was monitored throughout surgery, and the temperature was maintained at 37.0 ± 0.5°C using an adjustable heating pad during recovery from anesthesia.

### CES Electrode Implantation

After the TBI lesion, a CES electrode was implanted in the rat’s skull. Four burr holes were made using a dental drill (NE213, NSK-Nakanishi Inc, Tochigi, Japan) with a 1.5-mm burr for screw electrodes (1.6-mm-diameter pole; Plastics One, Inc., Roanoke, VA). According to the stereotaxic brain atlas of Paxinos and Watson, cortical electrodes were placed epidurally (*A* = + 4.0 mm, L = ± 2 mm for the frontal cortex; A = −3.6 mm, L = ± 4 mm for the parietal cortex; Paxinos and Watson, 2005; Figure 1B). All electrodes were inserted into a six-channel pedestal (MS363, Plastics One, Inc., Roanoke, VA, USA). The surgical incision was closed with three stitches. The screw electrodes and pedestal were secured to the skull surface with dental acrylic (Lang Dental Mfg., Wheeling, IL, USA; Figures 1C,D). Following TBI lesion and CES electrode implantation, the analgesia (Carprofen, 5 mg/kg; Pfizer Animal Health Inc., PA, USA) was administered subcutaneously every 24 h for 48 h postoperatively.

### CES Treatment and Experimental Design

**Figure 2:**
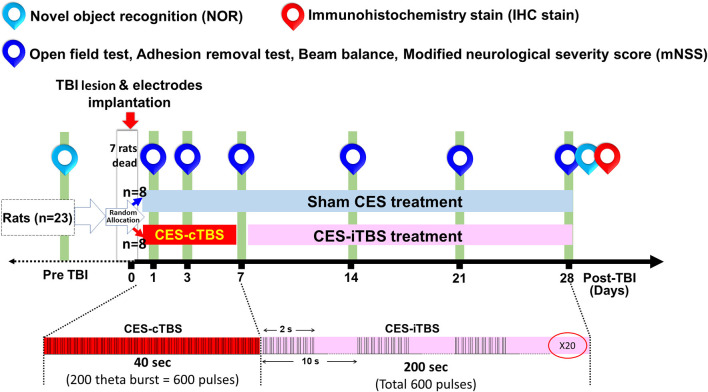
Experimental design for the long-term treatment effects of CES. The TBI rats in the sham CES group remained in conditions identical to those of the CES group for the same period of time. In the treatment group, CES was performed in 20 sessions over 28 successive days, a session/day for 5 consecutive days per week. The inhibition and facilitation protocols were applied under the acute and subacute stages, respectively. The open field, adhesion removal, beam balance, and modified neurological severity score (mNSS) tests were performed every week to investigate long-term treatment effects. The novel object recognition (NOR) test was measured at baseline and 28 days post TBI lesion to identify the function of short-term recognition memory.

Twenty-three rats were used for the present experiment. Following TBI lesion, a 30.4% (7/23) mortality rate was observed following weight-drop induced TBI. 16 TBI rats were utilized to observe the efficacy of CES treatment. Animals were randomly divided into the sham CES treatment group and CES treatment group (*n* = 8 in each group). For the stimulation protocol of CES, we applied a popular and specific rTMS paradigm, the continue theta-burst stimulation (TBS; cTBS) and intermittent theta-burst stimulation (iTBS) protocol, have been proposed for inducing more efficient long-term potentiation (LTP) or long-term depression (LTD)-like plasticity in the motor cortex beyond the short period of stimulation and lower intensity (Huang et al., 2005; Fitzgerald et al., 2006; Khedr et al., 2007). The basic pattern of cTBS or iTBS consisted of three pulses at 50 Hz and repeated every 200 ms (Figure 2). In the cTBS paradigm, the stimuli were given in a continuous train lasting 40 s (i.e., 600 bursts; Huang et al., 2005). The iTBS scheme was given in a 2-s train and repeated every 10 s for 20 cycles (190 s, total 600 pulses). The stimulus intensity was set at 80% resting motor threshold (RMT). The RMT was defined as the minimal intensity of CES required for eliciting minimal forelimb muscle twitches. Under the intensity at 80% RMT for CES treatment, no obvious muscle twitches were observed during CES treatment.

In the CES treatment group, the CES intervention protocols were divided into two stages: the acute stage and the chronic stage. In the acute stage (24 h-1 week post-TBI), the CES treatment protocol using continuous theta-burst stimulation (cTBS) was designed for the suppression of the hyperexcitability cascade, which may prevent or minimize some of the disabling consequences of TBI and have a potential therapeutic effect (Demirtas-Tatlidede et al., 2012; Villamar et al., 2012). In the chronic stages (>1 week), the CES parameter using intermittent theta-burst stimulation (iTBS) was set to modulate or increase brain plasticity, which could be useful to reduce functionally maladaptive changes to counter disability (Demirtas-Tatlidede et al., 2012). One day after the TBI lesion, the TBI animals in the CES group received the CES-cTBS protocol at an intensity of 80% RMT for 40 s daily for five consecutive days on the first 7 days. In the subacute and chronic stage, from 7 days to 28 days post-TBI, the CES-iTBS protocol (1 session/day, five consecutive days/week, pulse intensity = 80% of the RMT) was carried out to evoke neural facilitation (Figure 2). In the sham CES treatment group, the TBI rats also experienced the same CES protocol but did not receive any electrical stimulation at the same time points. All TBI rats were allowed to move freely during the CES or sham CES intervention. Behavioral tests, including open field locomotor activity tests, adhesion removal tests, beam walking, and mNSS, were performed at baseline (pre-TBI) and 1, 3, 7, 14, 21, and 28 days post-TBI lesion. For the cognitive measure in TBI rats, because the exploratory behavior could be influenced by the impairment of locomotor ability during the novel object recognition test, to avoid this potential confound, the novel object recognition test was assessed pre-lesion and at 28 days post-TBI lesion. Immunohistochemistry analysis was applied on day 28 post-TBI lesion to identify the changes in neural inflammation levels following CES treatment.

### Behavioral Tests

A well-trained examiner was blinded to the type of intervention and performed all examinations before and after sham or CES treatment. All experimental animals were trained and pretested for these tasks at least 3 days before TBI lesion to record the baseline level (as pre-TBI data). After habituation and training, all behavioral test sessions were performed at our set time points under the same environmental conditions. Behavioral tests measured the time-course changes in sensorimotor and cognitive functions associated with TBI, i.e., mNSS, adhesion removal tests for sensory function, beam walking tests for balance function, and novel object recognition tests for short-term recognition memory. There was at least a 4-h break between behavioral tests to avoid possible interference.

### Assessment of Motor Symptoms in TBI Rats

#### Beam-Walking Test

Balance and coordination were assessed by the beam-walking test (Dixon et al., 1987). Animals were pretrained to walk along the Plexiglas beam (120 cm long, 1.5 cm wide) toward their home cage at the opposite end. The latency of walking across within five testing trials after injury was calculated (Yu et al., 2016; Hsieh et al., 2017).

#### Adhesion-Removal Test

Sensory function was evaluated by the adhesion-removal test (Albertsmeier et al., 2007; Hsieh et al., 2017). Rats were familiarized with the environment. Two small dot stickers were attached to the bilateral forelimb. The removal duration was recorded.

#### Modified Neurological Severity Score (mNSS)

The mNSS is one of the most common neurological scales applied in animal studies of stroke. Severe TBI shares similar symptoms and pathology with stroke. The mNSS might also be a good test to evaluate the cortical functions of TBI rats. The mNSS includes a composite of balance, motor (muscle status and abnormal movement), sensory (visual, tactile, and proprioceptive), and reflex tests (Schaar et al., 2010; Hsieh et al., 2017).

#### short-term Recognition Memory

A novel object recognition (NOR) test was used to evaluate short-term recognition memory based on the tendency of rats to discriminate between familiar and new objects (Cheng et al., 2015). Before the acquisition phases, rats were habituated in the open field box for 10 min on day 3 and were then transferred to the home cage for 2 min. After that, the animals were placed back in the box for 10 min with the addition of two objects made of the same material placed in a symmetrical position. After 1 h, one of the objects was replaced with a novel object, and exploratory behavior was again analyzed for 10 min (day 3). The exploration duration was defined as sniffing, rearing on the object at a distance of less than 2 cm, or touching it with the nose (Zhang et al., 2019). The data was further analyzed as the discrimination index, which is defined as the exploration time at the novel object—the exploration time at the familiar object / the exploration time at the novel object + the exploration time at the familiar object (Aggleton et al., 2010; Antunes and Biala, 2012). After each session, the objects were cleaned with 75% ethanol to prevent odor recognition from impacting the testing results.

#### Locomotor Activity

The open-field test was applied to measure general locomotor activity. In this test, each rat was monitored in an open field black plexiglass arena (60 × 60 × 100 cm in dimension) by a video camera. The total distance traveled and the movement time of each animal was recorded within a 10 min testing period (Feng et al., 2020). Each trial was recorded and analyzed using the tracking system (Smart 3.0, Panlab, Harvard Apparatus, Barcelona, Spain). The testing area was thoroughly cleaned with 75% ethanol between each testing period for each rat to avoid odor interference in the test response.

### Immunohistochemistry Staining

After behavioral tests at 28 days post-lesion, TBI rats were sacrificed for glial fibrillary acidic protein (GFAP) staining. Briefly, brains were postfixed in a 4% paraformaldehyde fixative solution (PFA) and cytoprotected in 30% sucrose solution for 48 h at 4°C until the brain sank. The cerebral tissues between −3.00 and −3.36 mm to bregma were sectioned into coronal blocks at a thickness of 30 mm on a cryostat (Leica CM3050S Cryostat, FL, US), and the areas of the frontal cortex, corpus callosum, and hippocampus were selected. The sections were quenched with 0.3% H_2_O_2_/PBS for 10 min and 10% milk (Anchor Shape-up, New Zealand) for 1 h to block nonspecific antibodies and then incubated with rabbit primary anti-GFAP (1:1,000, AB7260, Millipore, USA) for 1 h at room temperature. After the sections were washed three times with PBS, they were incubated with the secondary anti-rabbit antibody (1:200, MP-7401, Vector Labs, USA) for 1 h at room temperature. The sections were developed by using a solution of 3.3-diaminobenzidine (DAB, SK-4105, Vector Labs, USA) for 5 min. Next, the sections were dehydrated in graded alcohols, cleared in xylene, and mounted with DPX. Mounted coronal sections were digitally imaged at 40x optical zoom (0.25 μM/pixel) using a digital pathology slide scanner (Aperio CS2, Leica Biosystems Inc. Buffalo Grove, IL, USA). For revealing the different expression levels of GFAP-positive cells between sham CES and CES rats, the higher magnification pictures were selected and captured from the Aperio ImageScope viewer software for further quantification and of GFAP-positive cells and observation of the morphology of GFAP-immunoreactive astrocytes. The consistent regions of interest for the frontal cortex, corpus callosum, and hippocampus were manually outlined according to the atlas of Paxinos and Watson (Paxinos and Watson, 2005). The obtained images with various degrees of GFAP expression were converted to binary (8-bit black-and-white) images. The binary threshold was determined to capture the GFAP-positive cells in the regions of interest while minimizing background staining and were kept constant for all images. The particle size used in particle analysis was set so that almost all astrocytes could be detected (Wakasa et al., 2009). The numbers of GFAP-positive cells in each region were counted by means of particle analysis under a computer-based image analysis system (Image-pro, Media Cybernetics, Bethesda, MD, USA) and then manually validated by two investigators in order to ensure the correct identification of immunoreactivity patterns. The density of GFAP-positive cells was calculated by individually counting the number of GFAP-positive cells within the region and was expressed as the mean numbers of cells per mm^2^ (cells/mm^2^) for further statistical analysis.

### Data Analysis

Data were analyzed using SPSS version 21.0 with the significance level set as *p* < 0.05 for each analysis. All data are presented as mean ± SEM. The effect of CES on the behavioral tests (i.e., beam-walking test, adhesion-removal test, mNSS, locomotor activity) was evaluated by a two-way repeated-measures analysis of variance (ANOVA) with the group (CES and sham CES treatment) as the between-subject factor and time (pre, every week after sham or CES treatment over 4 weeks) as a within-subject factor. For the short-term recognition memory, a two-way repeated measures ANOVA was used with two-way with the group (real CES and sham CES treatment) as the between-subject factor and time (pre and after sham or CES treatment over 4 weeks) as a within-subject factor. Unpaired *t*-tests were performed to compare groups at each time point when the main effect of the group was significant. Furthermore, the *post hoc* Fisher’s LSD tests were also used to compare time points on behavioral and immunohistochemical data.

## Results

### Effect of CES Treatment on Neurological Function in TBI Rats

**Figure 3:**
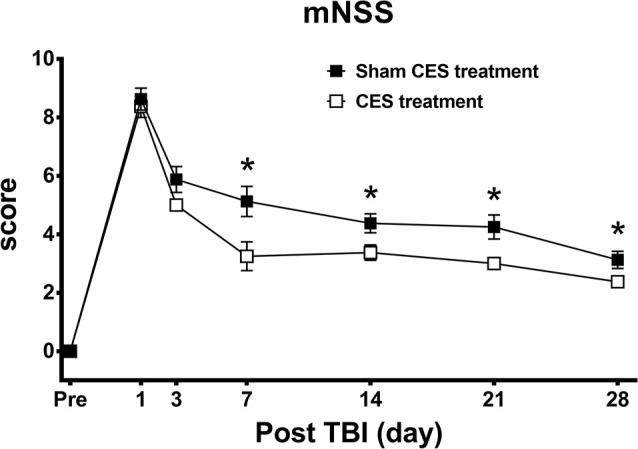
Effect of CES treatment on neurological function evaluated by mNSS. Values are expressed as the mean ± SEM. *p < 0.05, significant difference between the two groups.

The neurological function of rats was rated by the mNSS, which is a multifunctional evaluation scale that comprises balance, sensory, motor, and reflex tests. Repeated measures ANOVA identified significant main effects of time (*F*_6, 84_ = 148.373, *p* < 0.001) and group (*F*_1, 14_ = 11.738, *p* = 0.004) and a significant time × group interaction (*F*_6, 84_ = 2.154, *p* = 0.04). When compared with baseline value, subsequent* post hoc* Fisher’s LSD tests demonstrate that the mNSS score was significantly increased at day 1 after TBI lesion (*p* < 0.001) and remained maintained in the high level for up to 28 days after TBI lesion (all *p* < 0.001 in both groups). For the comparison of the mNSS score between two groups, *post hoc* *t*-tests between the two groups showed that the scores reached significant differences at day 7 (*p* = 0.02), day 14 (*p* = 0.031), day 21 (*p* = 0.015), and day 28 (*p* = 0.049) after TBI lesion (Figure 3).

### Effects of CES Treatment on Locomotor Dysfunction and Balance Function in TBI Rats

**Figure 4:**
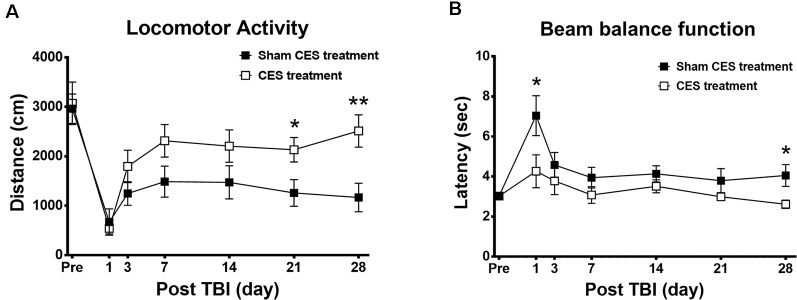
Effects of CES treatment on **(A)** locomotor activity and **(B)** balance assessed by the open field test and beam walking test, respectively. Values are expressed as the mean ± SEM. *p < 0.05, **p < 0.01, significant difference between the two groups.

In the open field test, the overall distance traveled was calculated to investigate the general locomotor activity between the CES group and the sham CES group following the TBI lesion. Repeated measures ANOVA indicated a significant main effect of time (*F*_6, 84_ = 21.010, *p* < 0.001) and a time × group interaction (*F*_6, 84_ = 2.575, *p* = 0.024) but not an effect of group (*F*_1, 14_ = 3.799, *p* = 0.072). When compared with baseline value, subsequent *post hoc* Fisher’s LSD tests show that locomotor activity was significantly decreased at day 1 after TBI lesion (*p* < 0.001) and remained maintained in the low level for up to 28 days after TBI lesion in the sham treatment group (all *p* < 0.001). However, in the CES treatment group, when compared with baseline value, *post hoc* tests show that the locomotor activity was significantly decreased on day 1 and day 3 after TBI lesion (*p* < 0.01). No significant differences were found between baseline and the time points of observation after 7 days post TBI lesion in the CES treatment group. For the comparison of locomotor activity between the two groups, *post hoc* *t*-tests between the two groups showed that the distance traveled in the open field test was significantly different at day 21 (*p* = 0.031) and day 28 (*p* = 0.008) after TBI (Figure 4A).

Furthermore, the beam walking test was applied to investigate the balance and motor coordination of the TBI rats with or without CES invention (Figure 4B). Repeated measures ANOVA identified significant main effects of time (*F*_6, 84_ = 6.874, *p* < 0.001) and group (*F*_1, 14_ = 6.844, *p* = 0.02) but not a time × group interaction (*F*_6, 84_ = 1.722, *p* = 0.126). When compared with baseline value, subsequent* post hoc* Fisher’s LSD tests show that latency to traverse the beam was significantly increased at day 1 after TBI lesion (*p* < 0.001). No significant differences were found between baseline and the time points of observation after 3 days post TBI lesion in the sham treatment group. For the comparison of beam balance function between two groups, *post hoc* *t*-tests between the two groups showed that the latency in the beam walking test was significantly different at day 1 (*p* = 0.049) and at day 28 (*p* = 0.039) after TBI (Figure 4B).

### Effect of CES Treatment on Sensory Function in TBI Rats

**Figure 5:**
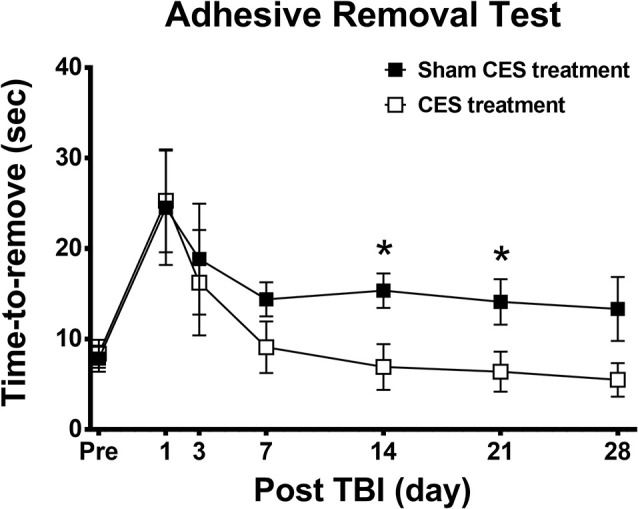
Effect of CES treatment on sensory function assessed by adhesive removal test. Values are expressed as the mean ± SEM. *p < 0.05, significant difference between the two groups.

The adhesive removal test was adopted in this study to observe sensory function in TBI rats. Repeated measures ANOVA applied to the removal time indicated significant main effects of time (*F*_6, 84_ = 7.012, *p* < 0.001) but not group (*F*_1, 14_ = 1.731, *p* = 0.209) or the time × group interaction (*F*_6, 84_ = 0.814, *p* = 0.562). When compared with baseline value, subsequent *post hoc* Fisher’s LSD tests show that sensory function was significantly decreased at day 1 after TBI lesion in both groups (*p* < 0.01). No significant differences were found between baseline and the time points of observation after 3 days post-TBI lesion in both groups (all *p* > 0.05). For the comparison of locomotor activity between the two groups, *post hoc* *t*-tests between the two groups showed that removal time showed a significant difference at day 14 (*p* = 0.019) and day 21 (*p* = 0.037) after TBI lesion (Figure 5).

### Effect of CES Treatment on Recognition Memory in TBI Rats

**Figure 6:**
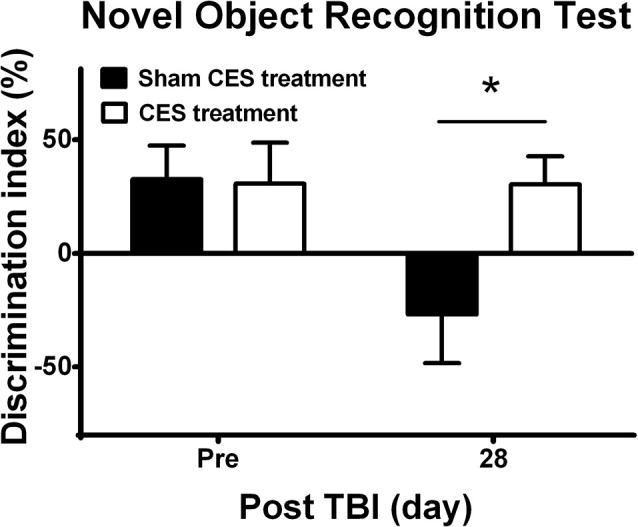
Effect of CES treatment on recognition memory evaluated by the novel object recognition test. Values are expressed as the mean ± SEM. *p < 0.05, significant difference between the two groups.

Novel object recognition was used to evaluate short-term recognition memory based on the tendency of rats to discriminate between familiar and new objects. Repeated measures ANOVA indicated a significant main effect of time (*F*_1, 14_ = 5.28, *p* = 0.038) and time × group interaction (*F*_1, 14_ = 5.15, *p* = 0.04) but not an effect of group (*F*_1, 14_ = 2.92, *p* = 0.109). Independent *t*-tests between the CES and sham CES groups showed that the discrimination index was significantly different at day 28 after TBI lesion (Figure 6; *t* = 2.67, *p* = 0.018).

### Effect of CES Intervention Assessed by Immunohistochemistry in TBI Rats

**Figure 7:**
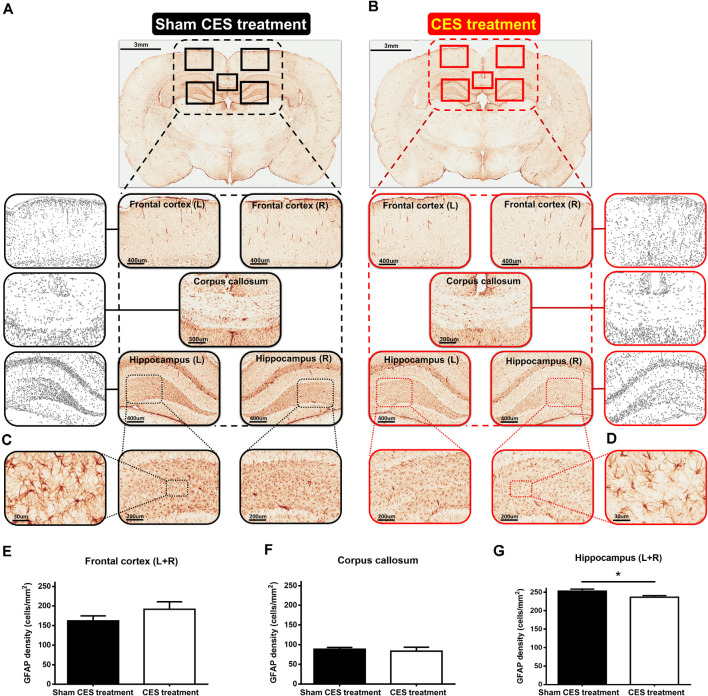
**(A,B)** Representative of glial fibrillary acidic protein (GFAP) immunostaining and example of GFAP images to a binary image in the frontal cortex (L + R, Magnification, ×50; Scale bars = 400 μm), corpus callosum (Magnification, ×50; Scale bars = 300 μm), and hippocampus (L + R, Magnification, ×50; Scale bars = 400 μm) in TBI rat with sham CES treatment and TBI rat with 4 weeks of CES treatment. **(C,D)** Representative views of the morphology of GFAP-positive cells in the hippocampus (Magnification, ×300; Scale bars = 30 μm). **(E–G)** The averaged data (L + R) of the density of GFAP-positive cells in the frontal cortex, corpus callosum and hippocampus in the sham CES treatment and CES-treated groups. *p < 0.05, significant difference between the two groups.

With regard to the effects of the 4-week CES intervention on GFAP positive cells, the results of GFAP immunohistochemistry in the frontal cortex, corpus callosum, and hippocampus are shown in Figure 7. The densities of GFAP-immunoreactive astrocytes in the frontal cortex and corpus callosum were similar between the two groups (Figures 7A,B,E,F). When compared with sham CES-treated rat, lower density of GFAP-positive cells in the hippocampus of CES-treated rat was found (Figures 7C,D). No morphological abnormalities or obvious hypertrophy in the GFAP-positive astrocytes were found between sham CES and CES rats. Independent *t*-tests between the CES and sham CES groups showed that the density of GFAP-positive cells was significantly lower in the CES treatment group compared with the sham CES group in the hippocampus (*p* = 0.033; Figure 7G).

## Discussion

In the current study, we applied a TBI rat model to investigate the effect of long-term CES treatment for 4 weeks on the TBI rats. We found that early and long-term CES intervention can ameliorate TBI-induced dysfunctions in sensorimotor and memory behavior. Moreover, the immunohistological results showed that long-term CES could ameliorate the TBI-induced elevations in GFAP in the hippocampus, suggesting that less central nervous system (CNS) damage was found in the CES treatment group. To date, the therapeutic efficacy and the underlying mechanisms of CES treatment for TBI remain unknown. An animal model would help provide more information on the benefits and underlying mechanism of CES treatment of TBI. Here, we performed several comprehensive and quantitative assessments of neurological severity score, sensory function, balance, and short-term memory, all of which are commonly affected in TBI patients, to identify the beneficial effects during and after 4 weeks of CES. After treatment with CES for 4 weeks, a clear improvement in all parameters of the mNSS score was observed in the CES treatment group. Moreover, compared with the sham CES treatment, CES treatment for 4 weeks ameliorated and delayed disease progression in the TBI rats. To the best of our knowledge, this is the first study to confirm the therapeutic effect of CES on several comprehensive motor and nonmotor functions in a TBI rat model. These data strengthen the growing amount of basic research and clinical literature on the efficacy of CES in TBI treatment.

For the design of the potential CES stimulation protocol, in this study, two theta-burst stimulation (TBS) protocols, intermittent TBS (iTBS) and continuous TBS (cTBS), were applied to induce long-term potentiation-like or long-term depression-like plasticity at specific time points during disease development (Hsieh et al., 2015a,b). With regard to the natural development pattern of neuropathological changes following TBI, several key molecular and biochemical processes have been identified in earlier studies. For example, following TBI, excessive glutamate accumulation is induced and causes NMDA-mediated glutamatergic excitotoxicity (Faden et al., 1989). Additionally, increased NMDA receptor activation results in neuronal and glial depolarization. Intracellular calcium overload induces further inflammation, mitochondrial dysfunction, and apoptosis (Katsura et al., 1994; Forster et al., 1999; Bramlett and Dietrich, 2004). Furthermore, overactive calcium levels may trigger calcium-induced calpain proteolysis of cytoskeletal proteins and subsequent cellular collapse (Bramlett and Dietrich, 2004). Cellular destruction may also result from increased oxidative stress due to mitochondrial dysfunction and increased neuronal and inducible nitric oxide synthase, enhancing the production of free radicals and lipid peroxidation (Forster et al., 1999; Syntichaki and Tavernarakis, 2003; Bramlett and Dietrich, 2004). Therefore, in this acute stage, we tried to apply the inhibitory theta burst protocol using CES-cTBS to suppress the hyperexcitability cascade and prevent or minimize some of the disabling consequences. In the subacute stage, based on an animal imaging study, GABA levels were found to be increased at 1–2 days post-TBI, as shown by magnetic resonance spectroscopy (Pascual et al., 2007). Additionally, TBI induces long-lasting working memory deficits associated with increased GABA levels, and administration of GABA antagonists restores memory function, suggesting that lasting deficits following TBI are associated with overreactive GABA-mediated inhibition (Kobori and Dash, 2006). Thus, in this stage (>1 week), we applied the facilitation protocol using the CES-iTBS scheme to counter GABAergic tone and increase neuronal excitability to further reduce functional deficits. Finally, in the chronic stage, the recovery process operating through synaptic reorganization may not be complete and adequate. This process may cause concentration of and damage to critical neural networks. The maladaptive plasticity of the brain may limit functional recovery and promote lasting disability. Therefore, in the chronic stages (1–4 weeks), we continued the facilitation protocol using the CES-iTBS scheme to increase brain plasticity and reduce the functionally maladaptive changes to counter disability. Based on current results, we found that the use of different neuromodulatory CES approaches at different stages after TBI could have the potential to reduce the sensorimotor and memory and promote functional recovery. However, although the time-course assessments of several functional behaviors have been made at different time points after CES treatment, we cannot clearly differentiate whether the long-term beneficial effects of CES come solely from CES-cTBS protocol during the acute stage of TBI or from the combination of both CES-cTBS and CES-iTBS protocols. It is one of the limitations in this study; hence a well-designed experiment is still needed in the future to prove the efficacy of CES in different stages after TBI and define the optimal CES protocols that maximize the functional recovery. Moreover, when compared with the sham treatment group, we found that early and long-term CES treatment can ameliorate TBI-induced dysfunctions. However, in this study, to avoid the possible interference between two evaluation time points and behavioral tests, we did not compare the pre-treatment values between two groups before CES or sham treatment. Thus, it cannot rule out the possibility of the severity difference between groups before the intervention. It is another limitation of this study. To eliminate this possible confounding factor, in addition to performing the standard procedures of TBI lesion, following TBI lesion, the TBI rats were randomly assigned to the control or treatment group before sham/CES treatment. These efforts could reduce the differences between groups before intervention.

CES is a cortical stimulation technique that has been used in the control of neuropathic pain (Fagundes-Pereyra et al., 2010; Alm and Dreimanis, 2013). Previous preclinical studies have shown that cortical stimulation can enhance neuronal plasticity and improve the functional performance in stroke rat model (Adkins-Muir and Jones, 2003). Furthermore, recent research suggests that CES can modulate motor cortical excitability *via* plasticity-like mechanisms (Hsieh et al., 2015a), and might have therapeutic potential for TBI (Adkins, 2015). In the current study, we found that 4 weeks of CES treatment of TBI rats improved the recovery of locomotor function. These results parallel the findings from another TBI animal study, showing that CES is effective for the recovery of motor function, spatial memory (Yu et al., 2018). In addition, it has been found that the 100 Hz CES combined with daily motor training for 9 weeks significantly improved forelimb motor performance (Jefferson et al., 2016), encouraging further research into its therapeutic potential. To date, it is still unclear what the optimal stimulation parameters of CES are, although they are currently under exploration *in vivo* or in human studies for TBI. A suitable disease animal model could help identify an effective stimulation protocol, including adjustment and optimization of frequency, polarity, and current level, allowing rapid screening and neurophysiological analysis in TBI animal studies. Future research is still needed to clarify the mechanisms of action of CES to explore and optimize CES protocols in TBI.

Histological investigation of GFAP staining was performed in both groups and revealed that CES ameliorated TBI-induced the upregulation of GFAP in the hippocampus in the group that received CES treatment compared with the group that received sham stimulation. GFAP is an astrocyte-specific intermediate filament component of the central nervous system (CNS) and is a common target used for observing the astroglial responses after TBI *in vivo* experiments (Myer et al., 2006; Chen et al., 2012). The astrocytes respond to TBI by pronounced changes in cell proliferation and cellular hypertrophy in the lesion site (Myer et al., 2006; Babaee et al., 2015; Cikriklar et al., 2016). In our earlier study using the same diffuse TBI rat model, the upregulation of GFAP has been found at day 1 and remained elevated for at least 1-week post TBI-lesion (Hsieh et al., 2017). In the current study, with the spontaneous recovery of functional behaviors, the GFAP-positive cells were found in the frontal cortex, corpus callosum, and hippocampus at day 28 after the TBI lesion, indicating sustained GFAP-positive astrocyte activation was still remarkably in the chronic stage after TBI. This finding was consistent with a previous TBI animal study using a controlled cortical impact rat model, showing GFAP-positive cells increased significantly in the cortex, corpus callosum, and hippocampus at 3 days, 14 days, and 28 days after TBI (Wang et al., 2018). Furthermore, when compared with the sham CES group, we found that long-term CES treatment significantly reduces the GFAP-positive astrocytes in the hippocampus at day 28 after the TBI lesion. The lower GFAP-positive astrocytes in the CES-treated group also support the beneficial effect on recognition memory and implies that the long-term CES treatment could be able to reduce the astroglial proliferation in the hippocampus, which may eventually alleviate the progressive deterioration of recognition memory in TBI rats. Although the CES electrodes were fixed on the skull and the electrical current was administered epidurally, the electric field could spread to 2–3 mm depth of rat’s brain (Asan et al., 2019). It indicates that the hippocampus could be stimulated and further induced the plastic or functional changes after CES. However, the detailed and precise mechanisms are still unclear. Further investigations are still needed to clarify the underlying mechanisms of the beneficial effects of CES.

## Conclusions

In conclusion, this study provides a clearer picture of the progressive symptom changes in induced TBI with or without CES treatment and documents the efficacy of CES in preventing several sensorimotor and memory dysfunctions. Future preclinical studies are still needed to further define the underlying mechanisms, leading to improved CES protocols for TBI.

## Data Availability Statement

The raw data supporting the conclusions of this article will be made available by the authors, without undue reservation.

## Ethics Statement

The animal study was reviewed and approved by the Institutional Animal Care and Use Committee, Chang Gung University IACUC Approval No.: CGU107-104, Period of Protocol: valid from October 01, 2018 to September 30, 2021.

## Author Contributions

C-WK, Y-ZH, and T-HH conceived and designed the experiments. C-WK, S-YC, P-KC, and T-HH performed the experiments. M-YC, H-HL, X-KH, C-WP, and C-YP provided the equipment. C-WK, H-HL, X-KH, and T-HH analyzed the data. C-WK, M-YC, and T-HH contributed to writing and editing the manuscript. All authors contributed to the article and approved the submitted version.

## Conflict of Interest

The authors declare that the research was conducted in the absence of any commercial or financial relationships that could be construed as a potential conflict of interest.
